# Supplementary material for: Research on technological innovation decision-making considering government subsidies and corporate reputation
Source: PLoS One. 2025 Jan 30;20(1):e0299667. doi: 10.1371/journal.pone.0299667 (PMC11781664; doi:10.1371/journal.pone.0299667)
Supplement: Attachment — Submitted filename: PONE-D-23-30152-R.docx [file pone.0299667.s001.docx]

**Research on technological innovation decision-making considering government subsidies and corporate reputation**

**Reviewer Comments:**

1. An organized conceptual framework that may direct the investigation is missing from the text. It is not obvious how business repute and government subsidies are factored into the decision-making process. The study's theoretical underpinnings and a precise research strategy should be established by the writers.
2. The literature review is insufficient and does not sufficiently highlight the holes that are currently in the area. To determine the importance of their findings, the writers must perform a more thorough examination of the pertinent literature.
3. The approach section is not specific enough. It is unclear how the data was gathered, which factors were taken into account, and which analytical techniques were employed. The sample size and data sources are also not disclosed. For the study to be believed, a solid technique is required.
4. It is difficult to assess the quality and validity of the data utilized in the study without knowledge of the data sources and the data gathering procedure. The data gathering process should be clearly described by the authors, along with steps taken to guarantee the quality and trustworthiness of the data.
5. If a quantitative analysis was done, the authors should go into great detail about the statistical procedures and tests that were utilized. This outlines the regression models and significance tests used in comprehensive detail. It is hard to judge the analysis's rigor in the absence of these data.
6. The research seems to show a causal relationship between business reputation, government subsidies, and choices about technological innovation. It is important to make it clear that correlation does not indicate causality. The research design's constraints and potential endogeneity problems should be discussed by the authors.
7. The discussion section ought to include more in-depth explanations of the results' implications. What are the practical ramifications for firms and decision-makers, and how do government subsidies and company reputation impact decision-making in technological innovation? The study ought to outline a more direct route from research to practical applications.
8. The authors' inclusion of specific recommendations for practitioners and policymakers based on their results would be advantageous. This would increase the research's applicability in real-world settings.
9. The paper needs to be better organized overall. It is challenging to follow the argument's logical progression. To make the document more coherent and clear, it should be reorganized.
10. The study's main conclusions, contributions, and limitations should be summed up in the conclusion. It ought to additionally emphasize the lines of inquiry for more study in this field.
11. To solve the conceptual, procedural, and analytical flaws, the study needs to be significantly revised. To be taken into consideration for publishing, the authors must present a more substantial and well-organized study that contribute to the body of knowledge on business reputation, government funding, and technological innovation decision-making.
