# Supplementary material for: Research on technological innovation decision-making considering government subsidies and corporate reputation
Source: PLoS One. 2025 Jan 30;20(1):e0299667. doi: 10.1371/journal.pone.0299667 (PMC11781664; doi:10.1371/journal.pone.0299667)
Supplement: Attachment — Submitted filename: Response to Reviewers.doc [file pone.0299667.s002.doc]

Dear reviewers and editors :

First of all, I would like to express my sincere appreciation to you for taking the time to read and modify my article. Thank you for your valuable suggestions. You have made comprehensive corrections to the structure, content, research methods and results of my paper. It plays a very important role in improving the quality of my thesis.

I carefully studied the reviewer 's comments and carefully revised the paper according to the recommendations. Reviewer comments are listed below in italics, with specific questions numbered. Our responses are given in normal font, and specific changes or additions to the manuscript are marked in red in the revised manuscript.

***Comment 1：An organized conceptual framework that may direct the investigation is missing from the text. It is not obvious how business repute and government subsidies are factored into the decision-making process. The study's theoretical underpinnings and a precise research strategy should be established by the writers.***

**Response: Thank you very much for the valuable suggestions of the reviewers ! We have improved it in the introduction of the article, and described in detail the process of corporate reputation and government subsidies being included in the decision-making. The modified part has been marked red in the introduction part.**

***Comment 2: The literature review is insufficient and does not sufficiently highlight the holes that are currently in the area. To determine the importance of their findings, the writers must perform a more thorough examination of the pertinent literature.***

**Response: Thanks for the valuable suggestions ! We re-combed and integrated the relevant literature, and gave the importance and innovation of this study. The above content has been shown in ' 2 Literature Review ' and has been marked red.**

***Comment 3: The approach section is not specific enough. It is unclear how the data was gathered, which factors were taken into account, and which analytical techniques were employed. The sample size and data sources are also not disclosed. For the study to be believed, a solid technique is required.***

**Response: Thank you very much for the valuable suggestions of the reviewers ! The data involved in the article is mainly the simulation part of the model. It is mainly through the assignment of key parameters to observe the changes of other variables in order to analyze the relevant trends.**

***Comment 4: It is difficult to assess the quality and validity of the data utilized in the study without knowledge of the data sources and the data gathering procedure. The data gathering process should be clearly described by the authors, along with steps taken to guarantee the quality and trustworthiness of the data.***

**Response: Thank you very much for the valuable suggestions of the reviewers ! Regarding the quality and effectiveness of the data, this paper refers to the processing methods of similar studies and assigns key parameters. The relevant references have been listed and marked red in the corresponding position of the ' 6 Numerical analysis ' section.**

***Comment 5: If a quantitative analysis was done, the authors should go into great detail about the statistical procedures and tests that were utilized. This outlines the regression models and significance tests used in comprehensive detail. It is hard to judge the analysis's rigor in the absence of these data.***

**Response: Thanks for the review expert's reminder! In this paper, the drawing software Matlab is used to analyze the analytical solutions obtained above. The reliability of the results is verified by analyzing the images. The specific modification has been specifically explained in the section of numerical analysis, and the relevant literature on the application of this method in similar studies is listed, including how to assign the key parameters and the range of the assignment. Corrections have been marked in red.**

***Comment 6: The research seems to show a causal relationship between business reputation, government subsidies, and choices about technological innovation. It is important to make it clear that correlation does not indicate causality. The research design's constraints and potential endogeneity problems should be discussed by the authors.***

**Response: Thanks for the guidance of reviewers ! This paper constructs a differential game model, and combines numerical simulation to confirm the impact of government subsidies and corporate reputation on technological innovation decision-making. It is summarized after Figure 6, and the relevant parts have been marked red.**

***Comment 7: The discussion section ought to include more in-depth explanations of the results' implications. What are the practical ramifications for firms and decision-makers, and how do government subsidies and company reputation impact decision-making in technological innovation? The study ought to outline a more direct route from research to practical applications.***

**Response: Thank the reviewers for their valuable suggestions ! We supplement the relevant content in the conclusion, and make a more in-depth explanation and description of the actual impact of the results on enterprises and governments, as well as how government subsidies and corporate reputation affect decision-making in technological innovation.**

***Comment 8: The authors' inclusion of specific recommendations for practitioners and policymakers based on their results would be advantageous. This would increase the research's applicability in real-world settings.***

**Response: Thanks to the reviewer 's kind reminder ! We have given relevant suggestions to the government and pharmaceutical companies, and reflected in the** **conclusion, the relevant content has been marked red.**

***Comment 9: The paper needs to be better organized overall. It is challenging to follow the argument's logical progression. To make the document more coherent and clear, it should be reorganized.***

**Response: Thank you for the professional advice of the reviewers ! We carefully review the writing context of the article, and give the logical arrangement of this article in the introduction. This part is reflected in the last paragraph of the introduction, which has been marked red.**

***Comment 10: The study's main conclusions, contributions, and limitations should be summed up in the conclusion. It ought to additionally emphasize the lines of inquiry for more study in this field.***

**Response: Thanks for your valuable suggestions ! We have re-combed the main conclusions, contributions and limitations of the article in the ' 7 conclusions ', and made a red mark.**

***Comment 11: To solve the conceptual, procedural, and analytical flaws, the study needs to be significantly revised. To be taken into consideration for publishing, the authors must present a more substantial and well-organized study that contribute to the body of knowledge on business reputation, government funding, and technological innovation decision-making.***

**Response: We sincerely appreciate the valuable comments! We re-examined the article and revised the comments of the reviewers in detail to make the article more logical and easy to read.**

Finally, thank you again for your guidance. If there are any other modifications we could make, we would like very much to modify them. We hope that under your guidance, we can complete this excellent paper, and we sincerely hope that the revised manuscript could be acceptable for you.
