# Supplementary material for: Research on technological innovation decision-making considering government subsidies and corporate reputation
Source: PLoS One. 2025 Jan 30;20(1):e0299667. doi: 10.1371/journal.pone.0299667 (PMC11781664; doi:10.1371/journal.pone.0299667)
Supplement: Attachment — Submitted filename: Plos One 1.docx [file pone.0299667.s003.docx]

The authors made diligent efforts to revise their paper. However, there are a few remaining changes that I believe should be addressed before we proceed. A few of my observations are as follows:

1. Your abstract does not highlight the specifics of your research or findings but contains too much background information. Some details of your research would be nice, for example, numbers addressing the sample, data, percentage improvement, etc. An abstract with some details helps show the impact of your research. This does not mean making the abstract longer! Shorter is better; remove some of the background material and add some details of your research. Again, it is good to provide some specifics (e.g., sample size, dataset size, numbers from results, etc.)
2. Avoid repetition in the manuscript (e.g., in the Abstract section, ‘………under three conditions: non-cooperative contract subsidized by the government, non-cooperative contract subsidized by the government, and cooperative coordination contract’).
3. Information about your dataset is missing. So, you need to explicitly provide a description of your dataset(s), in the manuscript, which is currently lacking. If the author(s) has provided only simulation part of the model, how the simulation part will be able to address the real world problem!
4. The author(s) must more clearly highlight their research’s theoretical and practical implications; it is unclear how this research differs from existing work. There should be an explicit section addressing the discussion of results and implications.
5. Comment 4,5&6 by previous reviewer, has not been addressed clearly. If the author(s) have used ‘Matlab’, describe the procedure thoroughly.
6. Provide a general discussion along with subheadings elaborating on 1) theoretical implications, 2) practical implications, 3) limitations and scope for future research, and 4) provide a short conclusion at the end of the manuscript.

Dear Editor,

Thank you for giving me the opportunity to review this article. Though the manuscript covers an interesting topic, I have found that the manuscript suffers gravely from conceptual and rational drafting errors. I have recommended major revisions after seeing the revised version.
