# Supplementary material for: Research on technological innovation decision-making considering government subsidies and corporate reputation
Source: PLoS One. 2025 Jan 30;20(1):e0299667. doi: 10.1371/journal.pone.0299667 (PMC11781664; doi:10.1371/journal.pone.0299667)
Supplement: Attachment — Submitted filename: PONE-D-23-30152R1-RR.docx [file pone.0299667.s004.docx]

**Research on technological innovation decision-making considering government subsidies and corporate reputation**

**Reviewer comments:**

I have provided you broad recommendations and remarks that reviewers might frequently make for a research paper on technological innovation decision-making taking government subsidies and business reputation into consideration, but I can't give you specifics on the substance of your work. Remember that these recommendations might need to be modified in light of the particulars of your paper:

Consider how well your literature review lays the groundwork for your study and how thorough it is. Make sure you have included pertinent research on government subsidies, company reputation, and technical innovation.

An important component is interpreting the findings. Talk about how your findings affect the way that decisions about technological innovation are made, taking into account both business reputation and government subsidies. Emphasise how your study is applicable in the real world.

Make sure that, in the context of technical innovation, your research successfully incorporates the two factors of government subsidies and business reputation. Talk about the interactions and effects these elements have on making decisions.

Give a concise summary of your research's management and policy implications. What conclusions can you draw from your research, and how can politicians or business implement these conclusions in real-world settings?

Clearly state how your study adds to the corpus of knowledge already in existence. How does your research contribute to our knowledge of how decisions about technological innovation are made?

Write a summary of your main conclusions and their wider ramifications. Summarise the importance of your findings and offer potential directions for further investigation.

Make sure your essay has a logical framework, is well-written, and is clear. Be mindful of your spelling, grammar, and formatting.
