# Supplementary material for: Research on technological innovation decision-making considering government subsidies and corporate reputation
Source: PLoS One. 2025 Jan 30;20(1):e0299667. doi: 10.1371/journal.pone.0299667 (PMC11781664; doi:10.1371/journal.pone.0299667)
Supplement: Attachment — Submitted filename: Response to Reviewers.doc [file pone.0299667.s005.doc]

Dear Editor and Reviewers,

We appreciate your professional comments on our article. As you are concerned, our article has several issues that need to be addressed. Based on your suggestions, we have made extensive changes to the previous manuscript! The reviewers ' comments are listed in italics below and numbered separately. Our reply is given in a normal font, and the specific revisions or additions to the manuscript are marked in red in the revised manuscript.

Response to reviewer 1 comments:

1. *Consider how well your literature review lays the groundwork for your study and how thorough it is. Make sure you have included pertinent research on government subsidies, company reputation, and technical innovation.*

We sincerely appreciated the valuable comments. We have checked the literature carefully and added more references on government subsidies, company reputation, and technical innovation into the Literature Review part in the revised manuscript.

2. *An important component is interpreting the findings. Talk about how your findings affect the way that decisions about technological innovation are made, taking into account both business reputation and government subsidies. Emphasize how your study is applicable in the real world.*

We sincerely appreciated the reviewers for their valuable suggestions! According to the results of the study, the Pareto improvement in system efficiency can be achieved under the government co-operative contract, when the level of technological innovation and the level of reputation of the firms are optimal compared to the non-cooperative contract without government subsidy and under government subsidy. The more sensitive the firm's reputation is to innovation efforts, the more it improves the firm's innovation level. In addition, the greater the social welfare brought about by the firm's innovation, the more it increases the government's subsidy coefficient. Therefore, enterprises should be oriented to consumer demand and maintain a good reputation level through continuous innovation, a good reputation level can attract government investment, government subsidies can share the innovation cost of enterprises, and under the government subsidy strategy, enterprises can achieve the improvement of the benefits of both government and enterprises through technological innovation. We attempted to use quantitative research methods to obtain the theoretical solutions of the three contract models, which to a certain extent solved the problem of practical implementation difficulties. Based on the explicit theoretical solution of this model, the government can optimise the model parameters and decide the type of contract according to the political demand and its own economic development status, etc. Similarly, the pharmaceutical enterprises can formulate a more accurate response strategy according to the current situation of the enterprise and the demand for innovation. In addition, we have tested the validity of the results through case studies based on the literature. More detailed modifications have been added to specific paragraphs in the text (**7 Conclusion**) and are marked in red.

3. *Make sure that, in the context of technical innovation, your research successfully incorporates the two factors of government subsidies and business reputation. Talk about the interactions and effects these elements have on making decisions.*

Thank you for your significant reminding. We carefully reviewed the article and revised and improved the relevant research details. In **6 Numerical analysis**, we added the content of the impact of the coefficient of technological innovation on reputation λ on the level of technological innovation K. And the effects of government subsidies and corporate reputation on firms' innovation decisions, as well as their interactions, are also fully analysed. Specific changes have been marked in red.

4. *Give a concise summary of your research's management and policy implications. What conclusions can you draw from your research, and how can politicians or business implement these conclusions in real-world settings?*

Thank you for your constructive advice. A total of three main findings were obtained from our study and based on the findings:

（1）When there is no government subsidy, the introduction of the non-cooperative contract of government subsidy, when 2A>B, the level of innovation efforts of pharmaceutical enterprises, the level of reputation, and the profits of both the government and the enterprise are improved, that is to say, the Pareto improvement of the government benefit, the enterprise benefit, and the overall benefit is realised.
（2）The more sensitive the enterprise's reputation is to the coefficient of technological innovation, the more it can improve the level of enterprise innovation.
（3）Under the government-enterprise co-operation pact, the level of innovation and reputation of firms is optimal, the overall efficiency of the system is maximised, and the efficiency of the government's innovation subsidy is significantly improved.

Reference recommendations are made for policy makers in government and pharmaceutical companies. Specific changes to the conclusions and policy recommendations are detailed in the‘**7 conclusion**’ section of the article and are marked in red.

5. *Clearly state how your study adds to the corpus of knowledge already in existence. How does your research contribute to our knowledge of how decisions about technological innovation are made?*

Thank you for pointing this out. The innovativeness of this paper is mainly reflected in the following aspects: firstly, a series of conduction relationships are established through the dissemination of reputation. A firm's technological innovation efforts affect the firm's reputation → the firm's reputation affects consumer demand → the demand further affects the firm's profit. By modelling this conduction relationship, a mathematical description of the problem is formed. Second, from the government's standpoint, the advantages and disadvantages of various participation methods are examined in order to reduce information asymmetry and obtain optimal subsidy efficiency. Thirdly, from the perspective of the firms, appropriate technological innovation decisions are made based on the subsidy strategy set by the government in order to maintain a good corporate reputation and further attract government support to stimulate corporate innovation, creating a virtuous circle and achieving sustainable development. More detailed explanations are given in the ‘**2 literature review**’ and in the‘**7.2 Theoretical and practical implications**’ of the ‘**7 conclusions** ’ section of the text and are marked in red

6. *Write a summary of your main conclusions and their wider ramifications. Summarize the importance of your findings and offer potential directions for further investigation.*

The valuable suggestions of the reviewers are highly appreciated! We have given the main contribution of the paper in the penultimate natural paragraph of the‘**2 Introduction**’ as well as in the‘**7 Conclusion**’ section respectively adding the main conclusions of the article, theoretical and practical implications and further research work in the future, which are marked in red.

7. *Make sure your essay has a logical framework, is well-written, and is clear. Be mindful of your spelling, grammar, and formatting.*

Thank you for your kind reminding. In order to make the content of the article clearer and more logical, we carefully reviewed the content of all chapters and made the language of the article smoother and more standardised through repeated revisions.

Response to reviewer 3 comments:

1. *Your abstract does not highlight the specifics of your research or findings but contains too much background information. Some details of your research would be nice, for example, numbers addressing the sample, data, percentage improvement, etc. An abstract with some details helps show the impact of your research. This does not mean making the abstract longer! Shorter is better; remove some of the background material and add some details of your research. Again, it is good to provide some specifics (e.g., sample size, dataset size, numbers from results, etc.)*

We think this is an excellent suggestion. We have re-written this part according to the reviewer’s suggestion. The specific changes can be seen in the **Abstract** section of the text and are marked in red.

2. *Avoid repetition in the manuscript (e.g., in the Abstract section, ‘………under three conditions: non-cooperative contract subsidized by the government, non-cooperative contract subsidized by the government, and cooperative coordination contract’).*

Thanks for your helpful suggestion. We have scrutinised the article and deleted the corresponding repetitions.

3. *Information about your dataset is missing. So, you need to explicitly provide a description of your dataset(s), in the manuscript, which is currently lacking. If the author(s) has provided only simulation part of the model, how the simulation part will be able to address the real world problem!*

Thanks for the kind reminders from the reviewing experts! In this paper, we construct a differential game model and solve it by using Hamilton-Jacobi equation, and in‘**6 Numerical analysis**’, we refer to the research results of the related literature as well as the real situation to set the parameter values in the model of this paper in a reasonable way. In fact, simulation can help us predict and model the behaviour and results in the real world, and by simulating the actual situation, we can better understand and solve the real problems. Although simulation cannot completely replace the reality, it can help us better understand the real problems and provide valuable references for the solution of real problems through parameter adjustment and model optimisation. There are a lot of scholars who use simulation for case analysis to solve real problems. These cases can prove the practicality and effectiveness of simulation. We list the relevant references in the text and mark them in red.

4. *The author(s) must more clearly highlight their research’s theoretical and practical implications; it is unclear how this research differs from existing work. There should be an explicit section addressing the discussion of results and implications.*

Thanks for your valuable suggestion. We have gone through the literature review by highlighting the differences between this study in ‘**2 Literature review**’ and existing studies and adding the theoretical and practical implications of this study in the ‘**7 Conclusion**’ section. All modifications are marked in red.

5. *Comment 4,5&6 by previous reviewer, has not been addressed clearly. If the author(s) have used ‘Matlab’, describe the procedure thoroughly.*

Thanks for your valuable counsel. In this paper, in order to show the details of the decision making results of this study more intuitively, numerical analysis is used to analyse the key indicators under different covenants. With the help of Matlab software, we use the parameter variables to be analysed as independent variables and dependent variables, and then substitute the values of the rest of the parameters to get the required equations, enter the equations by means of codes in the software, set certain intervals, and draw them with different lines, and then finally run all the codes to draw the corresponding images. By observing the images, the trend of key indicators can be analysed.

6. *Provide a general discussion along with subheadings elaborating on 1) theoretical implications, 2) practical implications, 3) limitations and scope for future research, and 4) provide a short conclusion at the end of the manuscript.*

Thank you for your valuable suggestions! In response to your suggestions we have added and improved the corresponding paragraphs in the text, added subheadings to the corresponding content, and marked the revised draft in red.

Thank you again for your valuable and constructive comments and suggestions on our manuscript. we sincerely hope that the revised manuscript could be acceptable for you.
